# Intrastriatal injection of preformed alpha-synuclein fibrils alters central and peripheral immune cell profiles in non-transgenic mice

**DOI:** 10.1186/s12974-019-1636-8

**Published:** 2019-12-03

**Authors:** Rachael H. Earls, Kelly B. Menees, Jaegwon Chung, James Barber, Claire-Anne Gutekunst, Manuel G. Hazim, Jae-Kyung Lee

**Affiliations:** 1 Department of Physiology and Pharmacology, University of Georgia College of Veterinary Medicine, 501 D.W. Brooks Drive, Athens, GA 30602 USA; 2 Department of Infectious Disease, University of Georgia College of Veterinary Medicine, Athens, GA 30602 USA; 3 Department of Neurosurgery, Emory University School of Medicine, Atlanta, GA 30322 USA

**Keywords:** Neuroinflammation, Synucleinopathies, Immune system, Parkinson’s disease, Animal models, Neurodegeneration, Lymphoid organs

## Abstract

Parkinson’s disease (PD) is characterized by the accumulation of alpha-synuclein (α-syn) inclusions, the major component of Lewy bodies. Extracellular α-syn aggregates act as a damage-associated molecular pattern (DAMP) and the presence of autoantibodies against α-syn species in the cerebrospinal fluid and the serum of PD patients implicate the involvement of innate and adaptive immune responses. In non-transgenic (Tg) mice, intrastriatal injection of preformed fibril (PFF) α-syn results in widespread pathologic α-syn inclusions in the CNS. While the PFF model has been broadly utilized to study the mechanistic relationship between α-syn transmission and other neuropathological phenotypes, the immune phenotypes in this model are not clearly demonstrated. This study aimed to characterize the immune phenotypes during pathologic α-syn propagation by utilizing PFF α-syn–injected non-tg mice. Here, we showed that pathologic α-syn inclusions are prevalent in various brain regions and the gut at 5 months post injection (p.i.), preceding the degeneration of dopaminergic neurons in substantia nigra (SN). We discovered a distinct inflammatory response involving both activation of microglia and astrocytes and infiltration of B, CD4+ T, CD8+ T, and natural killer cells in the brain at 5 months p.i. Moreover, PFF α-syn–injected mice display significant alterations in the frequency and number of leukocyte subsets in the spleen and lymph nodes with minimum alterations in the blood. Our data provide primary evidence that intracerebral-initiated synucleinopathies in non-tg mice alter immune cell profiles both in the CNS and peripheral lymphoid organs. Furthermore, our data provides support for utilizing this mouse model to assess the mechanistic connection between immune responses and synuclein pathology.

## Introduction

Parkinson’s disease (PD) is a devastating neurological disorder that affects over half a million people in the USA [1]. PD is pathologically characterized by the loss of dopaminergic (DA) neurons in the substantia nigra pars compacta (SNpc) and the accumulation of alpha-synuclein (α-syn) protein into Lewy bodies (LBs) [2]. While the etiology of PD is multifactorial, the role of inflammation has been deemed a significant contributor to neuropathology [3–5]. Early evidence revealed the presence of reactive microglia expressing Histocompatibility Leukocyte Antigen DR-Isotype (HLA-DR) in the SNpc of human post mortem brain tissue [6]. Furthermore, increased levels of inflammatory cytokines including interleukin 1-beta (IL-1β), interleukin-6 (IL-6), interferon-gamma (IFN-γ), and tumor necrosis factor-alpha (TNF-α) have been found within brain parenchyma and human cerebrospinal fluid (CSF) of PD patients [7–9]. Recent studies have shown that peripheral immune cells also play a critical role within the CNS [10–13] and the periphery [13, 14] in neurodegenerative disorders. In particular, CD4+ and CD8+ T cells were found in post mortem human brain tissues of PD patients, implicating involvement of the peripheral immune system during DA neurodegeneration [10].

It has been suggested that α-syn could be immunogenic in PD pathogenesis [15]. Extracellular α-syn induces microglial activation and increases proinflammatory cytokine gene expressions including TNF-α and C-X-C motif chemokine 10 (CXCL10) [16]. Oligomeric forms of α-syn have been detected in human CSF and blood plasma [17–21]. Importantly, the presence of autoantibodies against α-syn and its aggregates in the blood of PD patients [22] implicate the involvement of an adaptive immune response specific to α-syn species. Lately, the preformed fibril (PFF) α-syn model has been utilized as an animal model of PD because it exhibits many clinically relevant hallmarks of PD including dopaminergic cell loss, behavioral deficits, and synucleinopathies [23]. In this model, PFF α-syn acts as seeds for formation of the endogenous α-syn aggregates, which lead to propagation of pathology and subsequent neurodegeneration. While the PFF α-syn model has been widely utilized to explore the mechanistic relationship between α-syn transmission and other neurological phenotypes that are clinically relevant to PD [23]*,* their immune phenotypes during synuclein aggregation and propagation have not been fully characterized. PFF α-syn injection has resulted in neuroinflammation in the CNS in PFF α-syn rat models [12, 24] and in human α-syn overexpressing Tg mouse models [25, 26]; however, immune profiles in the CNS and the periphery in PFF α-syn models have never been interrogated in non-Tg mice. Here, we characterized immune phenotypes in the CNS and the periphery of PFF α-syn–injected non-Tg mice.

In this study, to interrogate immune responses mediated during α-syn aggregation and propagation, we analyzed immune responses at 5 months after PFF α-syn injection when the propagation of synuclein pathology is prevalent but DA neurodegeneration has not occurred to distinguish immune changes induced by neuronal degeneration. We showed that the transmission of pathological α-syn inclusions was not limited within the CNS, but also spread to the gut at 5 months post injection (p.i.). We demonstrated substantial changes in immune cell subsets in the brain and within peripheral lymphoid organs. Therefore, our study explored central and systemic changes in immune cell compositions during the prodromal stage of the PFF α-syn non-Tg mouse model of PD. Our results suggest that this model could be useful to interrogate the mechanistic link between immune responses and synuclein pathogenesis or other neuropathological phenotypes of PD.

## Methods

### Animals

C57BL/6J mice (8-week old males and females) were purchased from Jackson Laboratory. Experimental procedures involving the use of animals or animal tissue were performed in accordance with the NIH Guidelines for Animal Care and Use and approved by the Institutional Animal Care and Use Committee at The University of Georgia in Athens. Animals were housed in a climate-controlled facility with a 12-hour light/dark cycle.

### Recombinant α-syn expression and purification

Recombinant human α-syn (pET21a-α-syn, Addgene) was expressed in BL21(DE3)/RIL *Escherichia coli* and purified by size exclusion chromatography and Mono Q ion exchange chromatography [27]. To further remove endotoxin contamination, they were purified by High S support cation exchange chromatography as described [28, 29] and endotoxin test was done as described below.

### Endotoxin test

The endotoxin test was done according to the manufacturer’s protocol using Pierce^TM^ LAL Chromogenic Endotoxin Quantification kit (Thermo Fisher Scientific). Standards and protein samples were prepared in endotoxin free water and the absorbances were determined by a BMG FluoGoldStar plate reader. The final endotoxin test of purified human α-syn protein was less than 0.5 EU/mg.

### Fibril formation and seed preparation of recombinant α-syn protein

Fibrils were prepared by shaking purified monomer α-syn (5 mg/mL) in assembly buffer (10 mM Tris, 50 mM NaCl, pH 7.6) at 1100 rpm at 37 °C for 7 days. To generate PFF α-syn seeds, PFF α-syn were sonicated using a cup-horn ultrasonic water bath (QSONICA) (30% power, 1 h) at 4 °C immediately before the treatment in primary neurons or surgical injections. Fibril formation was confirmed using thioflavin T fluorimetry and EM imaging. The conformations of monomer α-syn, α-syn aggregtes, PFF α-syn, and PFF α-syn after sonication were confirmed by electron microscope (EM) images.

### Electron microscopy images

To collect EM images, a formvar, carbon-coated 400-mesh grid was floated onto a 40-μl drop of sample for 15 min. The grid was removed; excess sample was drained off with the edge of a filter paper and floated onto a drop of filtered 0.5% aqueous uranyl acetate (UA) for 30 s. Representative digital images were taken with an XR80M Wide-Angle Multi-Discipline Mid-Mount CCD Camera from Advanced Microscopy Techniques (AMT) at varying magnifications. The ImageJ software in the camera’s system was used to measure the fibers. The average size of sonicated PFF seed length was determined by values obtained from the analysis of aggregates in electron microscopy images.

### Primary hippocampal culture

Postnatal day 0-2 (P0-2) mouse pups were euthanized, brains dissected, and the hippocampus isolated. Following removal of the meninges, the hippocampal tissue was dissociated through enzymatic and mechanical means. Briefly, the tissue was incubated in a combination of dispase II (Roche), DNase I (Invitrogen), and papain (Sigma-Aldrich) for 15 min in a 37 °C, 5% CO_2_ incubator and then triturated with a fire polished glass pipet. Cells were then filtered through a 40-μm cell strainer. Cells were plated on poly-d-lysine coverslips in a 24-well plate at a density of 100,000 cells per well. Seven days following plating, neurons were treated with PBS or 1 μg/mL of sonicated PFF α-syn, non-sonicated PFF α-syn, or monomer α-syn. Seven days later neurons were fixed with 4% paraformaldehyde (PFA).

### Immunocytochemistry

Following fixation with 4% PFA, primary hippocampal neurons were incubated with primary antibodies against MAP2 (Invitrogen) (1:1000), p-α-syn (Abcam) (1:200), and Syn204 (Cell Signaling) (1:100) overnight. Cells were then incubated with Alexa fluor 647-, Alexa fluor 488-, and Alexa fluor 594-conjugated secondary antibodies (Invitrogen) (1:1000) for 2 h at RT. The coverslips were then mounted onto microscope slides with Fluoroshield Mounting Medium with DAPI (Abcam). Images were taken on a Nikon A1R confocal microscope (Nikon Eclips Ei-E inverted microscope).

### Stereotaxic surgery

Mice were anesthetized with Ketamine (100 mg/kg)/xylazine (10 mg/kg) and placed in a stereotaxic frame (KOPF) with ear bars. Animals received a unilateral injection of human PFF α-syn seeds or monomer (5 μg in 1 μl) into the right striatum using the following stereotaxic coordinates relative to bregma and the dural surface; AP, + 0.3 mm; ML, + 2.3 mm; DV, − 3.5 mm at the rate of 0.2 μl/min using a 29-gauge needle. Postoperatively and on the following 3 days, animals received subcutaneous injections of analgesics and were monitored closely for any signs of pain and discomfort.

### Immunohistochemistry

Mice were euthanized and perfused with ice-cold glucose-containing buffered saline followed by 4% PFA for fixation. The brains and intestines were removed and fixed in 4% PFA for 24 h followed by cryopreservation in 30% sucrose solution. Brains were coronally sectioned into 40-μm thickness and intestinal tissue was sectioned at 10-μm thickness. For bright field immunohistochemistry, sections were quenched in 3% hydrogen peroxide (H_2_O_2_) for 1 h and blocked in 10% normal serum for 1 h. Following blocking, sections were immunostained with anti-Tyrosine Hydroxylase (TH) (Chemicon/Millipore) or phospho-serine129 α-syn (p-α-syn) (Abcam, Ab51253, 1:25,000 dilution) and incubated overnight. Following washes, sections were incubated with the appropriate biotinylated secondary antibody followed by 1-h incubation with Vector ABC standard detection kit (Vector Laboratories). TH and p-α-syn staining was visualized by development in 3,3′ diaminobenzidine. For immunofluorescence, brain and intestinal sections were fixed for an additional 15 min in 4% PFA. Sections were incubated in 0.2-M glycine for 30 min to quench autofluorescence and permeabilized for 30 min in TBS containing 0.2% Triton X-100, followed by blocking for 1 h in 1% normal serum in TBS. Sections were incubated in primary antibodies including p-α-syn (Abcam), anti-TH (Chemicon/Millipore), anti-ionized calcium-binding adaptor molecule 1 (Iba-1) (Wako), anti-MHC-II (eBioscience), anti-glial fibrillary acidic protein (GFAP) (Thermo Fisher Scientific), and anti-microtubule-associated protein 2 (MAP-2) (Thermo Fisher Scientific) followed by the appropriate Alexa Fluor 488- or Alexa Fluor 594-conjugated secondary antibodies (Thermo Fisher Scientific).

### Proteinase-K digestion

A subset of free-floating sections from the striatum was treated with 10 μg/mL proteinase-K for 20 min at room temperature, followed by three washes in TBS and three washes in TBS containing 0.2% Triton X-100. Sections were then processed for p-α-syn (Abcam, Ab51253, 1:25,000 dilution) immunohistochemistry (IHC) as described above.

### Confocal microscopy

Images of MHC-II, TH, Iba-1, and GFAP were acquired using a Nikon A1R Confocal microscope. Images (12 bits/channel) and optical sectioning for a Z-series were obtained using a CFI Plan APO VC 60X Oil NA 1.4 WD 0.13 mm objective lens with 8 slices at 1.804-μm intervals at a sample speed of 21.6 μs/pixel. Z-series images of p-α-syn and MAP-2 were acquired using a CFI Plan APO VC 40X Oil NA 1.4 WD 0.13-mm objective lens with 6 slices at 1.370-μm intervals. Image analysis was conducted using NIS-Elements AR analysis 4.00.12 64-bit software. Colocalization analysis was conducted on a single slice and then presented as a maximum intensity projection.

### Image J analysis

Densitometric analysis of p-α-syn positive staining was performed using the freeware NIHImageJ1.43 software (National Institute of Health) in the striatum, SNpc, primary motor cortex, and hippocampus. Images were acquired through a Zeiss Axio Scope A1 microscope. Up to four images were captured at 20 × or 40 × for each section within each region of interest (ROI), with four 40-μm sections per animal. Measurements were carried out in a blind fashion. The average optical density (O.D.) for p-α-syn positive staining was calculated on the lesioned side of the brain, and data was plotted as the average O.D. value for each animal (*n* = 4–6 animals per experimental group/4 sections per animal). Quantification of immunoreactivity for the levels of astrogliosis and microgliosis were done using ImageJ software to measure O.D. of GFAP and Iba-1 staining within the SNpc. The data are presented as average immunoreactive area on the lesioned side of the brain (*n* = 3–4 animals per experimental group).

### Stereological analysis

To estimate the total dopaminergic neuron number within the SNpc, we used unbiased stereology using the Cavalieri method. This was performed using a Leica DM2500 light microscope in conjunction with Stereologer Version 3.0 CP—Version 2. Injected and non-injected sides were analyzed separately, as identified using a mark made prior to the cutting of the sections. The person performing the counts was blinded to the experimental conditions. For each SNpc, 40-μm coronal sections spanning the entire SNpc and every fourth section were used for the count for a total of eight sections per SNpc separated by 120 μm. The area of SNpc was outlined at 5 × according to the Paxinos and Franklin atlas [30], while the TH neurons were counted at 40 ×. The parameters used for optical dissection are as follows: frame size (25% of screen height^2^ or 2425 μm^2^), frame area (25% of screen height^2^ or 2425 μm^2^), frame height (20 μm), guard height (2 μm), and frame spacing (150 μm). The results displayed an average coefficient of error (CE) of 0.1252 and an average slice thickness of 23.3813 μm.

### Mononuclear cell isolation from the CNS

Mononuclear cells from the brain were isolated from the mouse as described previously [31] 5 months after stereotaxic injection of α-syn PFF seeds or monomers. Briefly, mice were sacrificed with an overdose of anesthetic and perfused with glucose-containing buffered saline. The brains were removed, minced with a scalpel, and then incubated in 3 mL of 2 × enzymatic dissociation medium containing sterile filtered DNase1 (1 μL/mL, Invitrogen), Dispase II (1.2 U/mL, Roche), and Papain (1 mg/mL, Sigma-Aldrich) dissolved in DMEM/F12 (Invitrogen) at 37 °C with continuous agitation for 20 min. After incubation, tissues were rinsed with PBS three times, then dissociated with polished Pasteur pipettes. Cells were resuspended in 4 mL of 37% isotonic percoll (Sigma), layered on a 30:37:70 percoll gradient, and centrifuged for 30 min at 500×*g* with no brake. Mononuclear cells were obtained from the 37:70% interface, washed twice in 1 × HBSS, and were counted and prepared for flow cytometry.

### Immune cell isolation from spleen, lymph node, and blood

The spleen and inguinal lymph nodes were collected from each mouse 5 months after stereotaxic injection of α-syn PFF seeds or monomers. Single cell suspension was prepared by mincing and filtering through a 70-μm cell strainer (Corning). Splenic red blood cells were lysed by resuspension of cell pellet in 5 mL of ACK lysing buffer (QualityBiological) for 5 min. Cells were counted and 2 million cells were used for flow cytometry. Blood was collected from atrial puncture and immediately transferred into EDTA Vacutainer collection tubes (BD) and mixed. EDTA-treated blood was then transferred into 50-mL conical tubes and red blood cells were lysed with 5 mL of ACK lysing buffer (QualityBiological) for 5 min and quenched with 1× HBSS. Cells were counted and 2 million cells were used for flow cytometry.

### Flow cytometry

Cells were suspended with FACS buffer (1 mM EDTA, 0.01% sodium azide, 0.1% bovine serum albumin (BSA), 0.02 M phosphate, 0.15 M NaCl, pH 7.2) and then stained for 20 min with anti-FcR/anti-CD16+CD32/Fc Block (eBioscience) and fluorophore-conjugated antibodies: anti-CD45-PerCP Cy5.5 (BioLegend), anti-Ly6G-AF700 (Thermo Fisher Scientific), anti-CD19-APC (BioLegend), anti-CD11b-PE (BioLegend), anti-NK1.1-PE Cy7 (BioLegend), anti-TCR-beta-Pac Blue (BioLegend), anti-CD4-FITC (BioLegend), and anti-CD8-APC/Cy7 (BioLegend). After staining, cells were washed and then fixed with 1% PFA for 30 min. After fixation, cells were washed three times with 200-μl FACS buffer, and then 50 μl of 123count eBeads Counting beads (Thermo Fisher Scientific) were added to allow for quantification of total number of immune cell subtypes following manufacturer’s instructions. Data were acquired on a LSRII instrument (BD Biosciences). Analysis was performed using FlowJo software, version 10.0.8.

### Gating strategy

Cells isolated from the CNS, spleen, inguinal LN, and blood were gated first on a forward (FSC) and side scatter (SSC), then total CD45 positive leukocytes were gated. This gating strategy allows for the selection of all immune cells (resident and peripheral) while eliminating doublets from analysis. TCR-β positive and CD19 negative T cells were gated from CD45+ parent population. NK 1.1 positive NK cells, Ly6G positive neutrophil, and CD11b positive monocytes were gated from non-B and non-T cells. For the cells from the CNS, resting microglia (CD11b+CD45^low^) and activated microglia (CD11b+CD45^high^) were gated from CD11b positive cells.

### Multiplex cytokine analysis

Serum was analyzed for chemokines and cytokines (IFN-γ, IL-1β, IL-2, IL-4, IL-5, IL-6, KC (CXCL1), IL-10, IL-12p70, and TNF-α) using a multiplexed immunoassay per the manufacturer’s instructions (Meso Scale Discovery, Rockville, MD).

### Statistical analysis

Statistical analysis and graphs were generated using GraphPad Prism 6 software. Student's *t* test was used for comparison between two groups. Significance was accepted at *p* values < 0.05 and data was presented as mean ± standard error of the mean (SEM) *p < 0.05, **p < 0.01, ***p < 0.001.

## Results

### Sonicated human preformed fibril α-syn seeds induce endogenous mouse α-syn pathology in *in vitro* primary mouse neurons

**Fig. 1:**
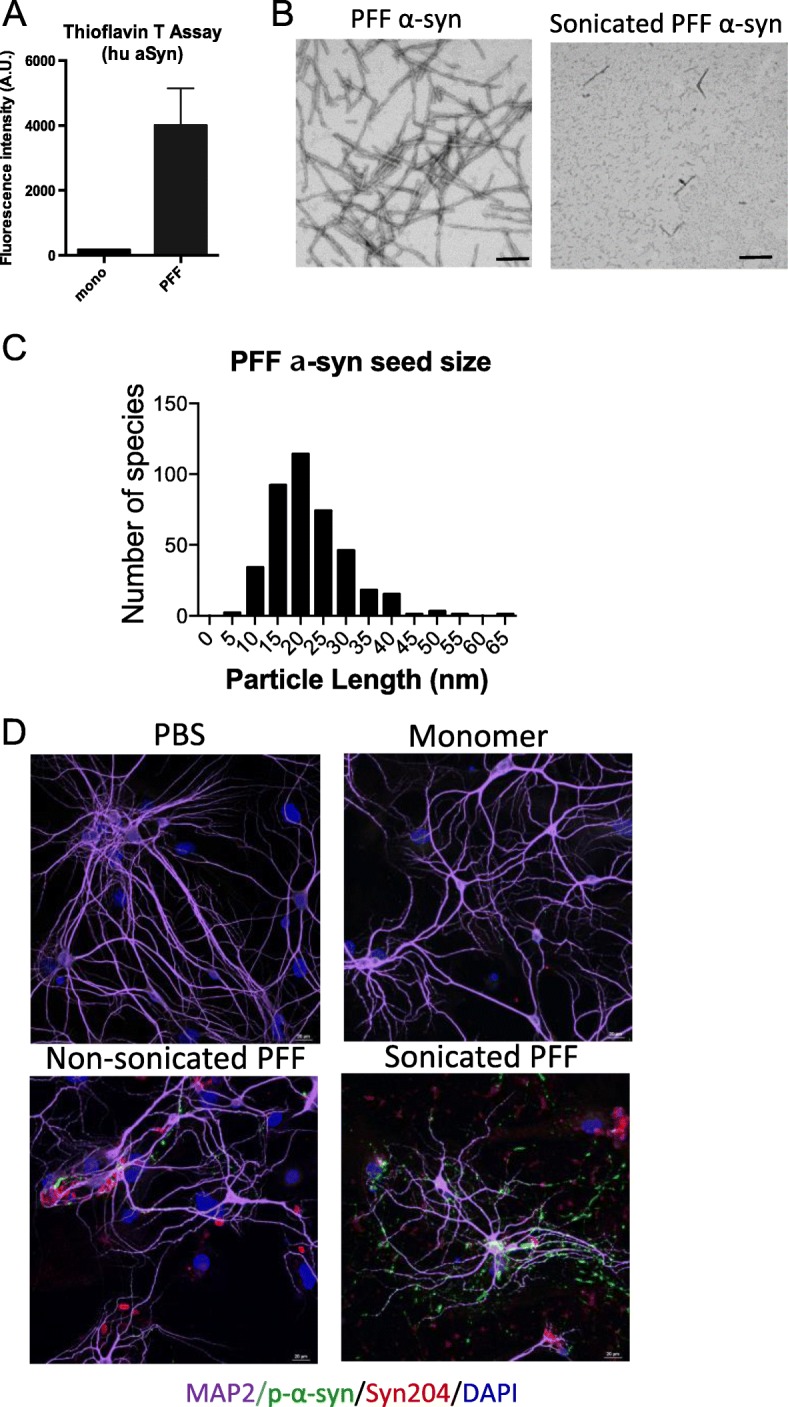
Preparation of human preformed fibrils (PFF) α-syn and validation of endogenous mouse α-syn inclusions in in vitro mouse primary neurons. **a** Thioflavin T assay showed the conversion of purified monomeric α-syn into PFF α-syn. **b** Representative electron microscopy images of PFF α-syn (left) and sonicated PFF α-syn (right). **c** Distribution of α-syn PFF length after sonication as determined by values obtained from the analysis of aggregates in electron microscopy images. Mean length of mouse PFF seeds are 21.74 nm ± 0.41 (*n* = 400). **d** Immunocytochemistry images of primary mouse neurons using antibodies against p-α-syn (green), MAP-2 (purple), and human α-syn Syn204 (red) to confirm PFF α-syn seeding and transduction capacity. On day 7 of neuronal culture, neurons were treated with PBS, 1 μg/mL of monomer α-syn, non-sonicated PFF α-syn, or sonicated PFF α-syn for 7 days, then were fixed on day 14. Scale bars are 20 μm

In this study, we utilized human preformed fibril (PFF) α-syn seeds to recruit endogenous murine α-syn and induce neurotoxicity *in vivo* [23]. We generated recombinant human α-syn as described [27]. As we aim to characterize immune responses in this study, the presence of endotoxin (Lipopolysaccharides) during the generation of recombinant α-syn could substantially affect outcomes. Therefore, we have carefully removed endotoxin contamination by processing α-syn preparation through additional two rounds of cation exchange steps as described [28, 29]. We confirmed that the final endotoxin amount of human α-syn preparation was less than 0.5 EU/mg. Furthermore, we used monomer α-syn as a control, which was the initial material used to generate PFF α-syn, in order to compare immune phenotypes with PFF α-syn–injected mice. We assembled fibrils as described in the methods section and confirmed conformation by thioflavin T assay and transmission electron microscopy (TEM) (Fig. 1a and b).

Prior to use, PFF α-syn was sonicated and the conformation and the average size of sonicated PFF α-syn were measured by TEM images (Fig. 1b). We have confirmed that the average size of sonicated PFF α-syn was 21.74 nm ± 0.41 (SEM) (Fig. 1c). We validated the seeding and transduction capacities of human PFF α-syn in primary mouse hippocampal neurons as previously reported [32]. We showed that sonicated PFF α-syn but not PBS, monomer α-syn, or PFF α-syn were able to induce p-α-syn inclusions in mouse primary neurons. Moreover, immunocytochemistry was performed utilizing mAb Syn204 (red), that specifically recognizes human α-syn and p-α-syn (green), and no colocalization was observed. This indicates that human PFF α-syn recruited endogenous mouse α-syn to form p-α-syn inclusions in primary mouse hippocampal culture (Fig. 1d).

### Unilateral intrastriatal injection of PFF α-syn induces Lewy body–like pathology in non-tg mice

**Fig. 2:**
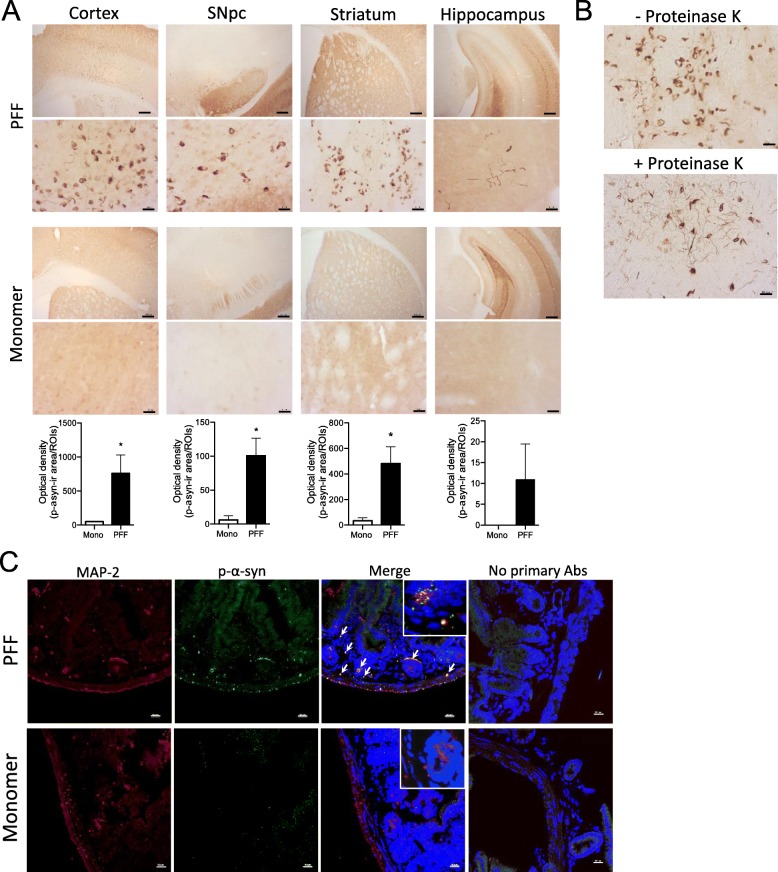
Validation of α-syn pathology in the CNS and peripheral gut tissue of C57BL/6 J mice. Stereotaxic injection of 5 μg human PFF α-syn or monomer α-syn into striatum (AP+0.3, ML+2.3, DV−3.5) of C57BL/6 mice was performed. Mice were sacrificed at 5 months p.i. (*n* = 5–6 animals/group). **a** Representative immunohistochemical images in the primary motor cortex, SNpc, striatum, and hippocampus of C57BL/6J mice. Scale bars represent 10 μm (40 × high magnification images) and 100 μm (5 × low magnification images). Graphs represent optical density (O.D.) of positive p-α-syn in the ipsilateral primary motor cortex, SNpc, striatum, and hippocampus (*n* = 4–6 per group). **b** Representative immunohistochemical images of α-syn in the striatum with or without proteinase-K treatment (10 μg/mL). Scale bars represent 10 μm. **c** Representative immunohistological images of p-α-syn and MAP-2 (neuronal marker) positive staining in the small intestines. Nuclear staining with DAPI shown in blue. Scale bar represents 20 μm**.** Asterisks denote significant differences with *p < 0.05, comparing PFF α-syn and monomer α-syn treated animals, according to Student’s *t* test

To induce α-syn pathology in non-tg mice, we performed stereotaxic surgeries to inject 5 μg (in 1 μl) of PFF α-syn or monomeric α-syn as a control in the striatum of C57BL/6 mice. In this model, PFF α-syn seeds are taken up by terminals within the striatum and transported through the nigrostriatal pathway back to the substantia nigra region where they act as a template and promote endogenous murine α-syn to accumulate into misfolded phosphorylated pathological aggregates [23]. To confirm the formation of pathological α-syn inclusions, we performed IHC analysis to quantitate pathological phosphorylated α-syn at serine 129 (p-α-syn) inclusions within the primary motor cortex, SNpc, striatum, and hippocampus at 5 months post injection (p.i.). Robust p-α-syn positive inclusions were present in the SNpc and striatum of PFF α-syn but not monomer α-syn–injected mice (Fig. 2a), indicating that intrastriatal PFF α-syn injection induced propagation of α-syn pathology throughout the nigrostriatal pathway. Furthermore, p-α-syn inclusions were found in the primary motor cortex and the hippocampus (Fig. 2a) confirming that LB-like inclusions propagate into different brain regions at 5 months p.i. as previously reported [23]. P-α-syn inclusions in the striatum 5 months p.i. displayed LB-like characteristics [33] including resistance to proteinase-K digestion (Fig. 2b). Recently, brain to stomach transfer of human α-syn has been demonstrated in Sprague Dawley rats 6 months after stereotaxic inoculation in the SNpc [34]. Furthermore, PFF inoculation into the gastric wall of C57BL/6J mice led to LB-like aggregates in the dorsal motor nucleus 45 days p.i [35]. To determine if p-α-syn inclusions were present within the ENS (small intestine) of our model, we performed IHC analysis using antibodies for p-α-syn and MAP-2 (a neuronal marker). We observed that PFF α-syn–injected mice displayed positive p-α-syn staining within the ENS at 5 months p.i. that was not seen in monomer α-syn–injected control animals (Fig. 2c).

### Unilateral intrastriatal injection of PFF α-syn induces microglial activation and astrogliosis preceding dopaminergic neuronal degeneration in the SNpc

**Fig. 3:**
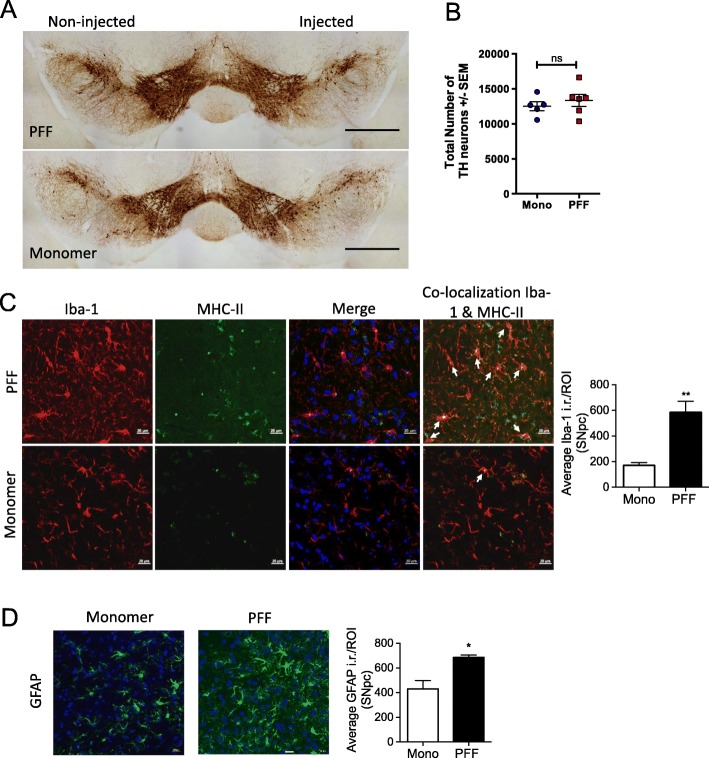
Unilateral intrastriatal injection of PFF α-syn induces microglial activation and astrogliosis preceding DA neuronal degeneration in the SNpc. C57BL/6J mice stereotaxically injected with PFF α-syn seeds or α-syn monomers. Immunohistochemical analyses were performed at 5 months p.i. (*n* = 5–6 per group). **a** Representative immunohistochemical images staining of TH in the SNpc. Scale bars represent 1 mm. **b** Stereological analysis of TH-positive neurons to evaluate total number of TH neurons in the SNpc. Graph shows mean ± SEM. **c** Representative immunohistochemical images of Iba-1 and MHC-II double staining in SNpc. Nuclear staining with DAPI shown in blue. Scale bars represent 20 μm. Graph represents optical density (O.D.) of positive Iba-1 staining in the ipsilateral SNpc (*n* = 3–4 per group). **d** Representative immunohistochemical images of GFAP staining in SNpc. Nuclear staining with DAPI shown in blue. Scale bars represent 20 μm. Graph represents O.D. of positive GFAP staining in the ipsilateral SNpc (*n* = 3–4 per group). Data represent mean ± SEM. *p < 0.05, **p < 0.01, ***p < 0.001, n.s.; not significant, Student’s *t* test

LB formation has been shown to be associated with DA neuronal degeneration [23]. Previously, unilateral intrastriatal injections of PFF α-syn in rat and mouse resulted in nigrostriatal degeneration around 6 months post injection [23, 24]. Our study aimed to characterize immune phenotypes that are associated with the formation and propagation of α-syn inclusions; therefore, we decided to quantify immune responses at 5 months prior to DA neuronal degeneration. To determine DA neuronal degeneration in PFF α-syn–injected mice, we performed IHC using an antibody against TH and conducted unbiased stereological cell counting to quantify TH-positive neurons in the SNpc. At 5 months, p.i. we did not observe any changes in TH-positive neuron number in PFF α-syn group compared with monomer α-syn–injected group (Fig. 3a and b).

Based on the robust positive p-α-syn staining in PFF α-syn treated animals, we examined the inflammatory responses within the SNpc. To characterize microglial activation, we double-labeled for Iba-1 and MHC-II to determine microglial activation, as MHC-II expression on activated microglia has been shown to be associated with proinflammatory cytokine release of TNF-α, IL-1β, and CD80 [36, 37]. Moreover, the levels of MHC-II were increased in the rat brain with LB-like inclusions [24]. Double labeling of Iba-1 and MHC-II confirmed the identity of MHC-II immunoreactive (i.r.) cells on microglia; furthermore, the i.r. area of Iba-1 positive cells was increased in the SNpc in mice injected with PFF α-syn compared to those with monomer α-syn, (Fig. 3c) indicating exacerbated inflammation in the PFF α-syn treated mice brains. In PD brains, inflammatory responses have been shown to be mediated by astroglial cells [38] as well. Based on the increased presence of MHC-II immunoreactive microglia and p-α-syn positive inclusions of PFF α-syn treated mice, we examined reactive astrogliosis. Reactive astrogliosis is characterized by increased expression levels of GFAP [5]. Optical density (O.D.) analysis of i.r. area of GFAP positive cells revealed increased astrogliosis in the SNpc of PFF α-syn treated mice compared with monomer α-syn (Fig. 3d). These data indicate that unilateral injection of PFF α-syn induces neuroinflammation within the CNS at 5 months p.i.

### Increased peripheral immune cell infiltrations in the CNS parenchyma in the PFF α-syn–injected mouse brain

**Fig. 4:**
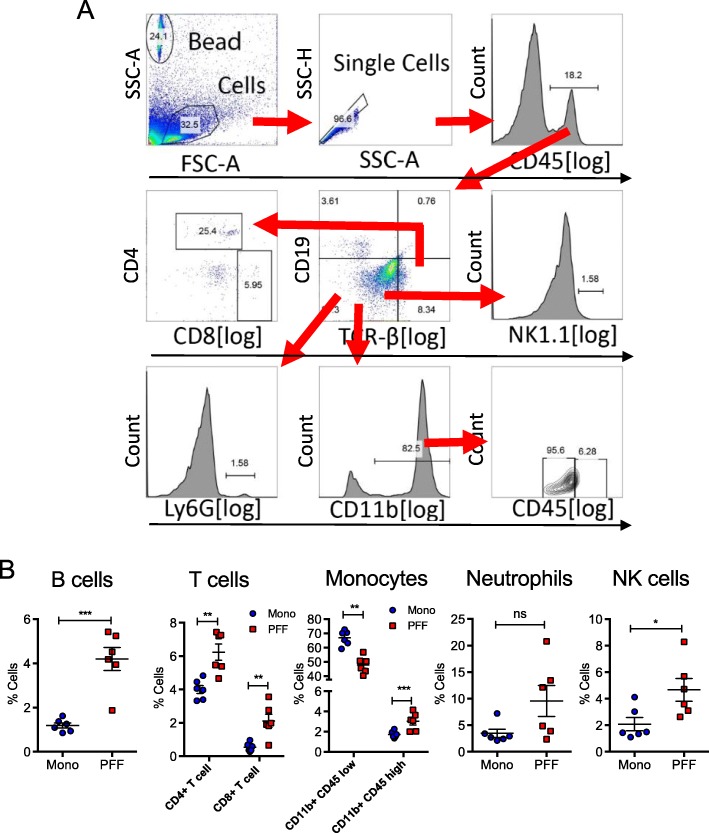
Peripheral immune cells infiltrate in the CNS parenchyma in the PFF α-syn mice. Mononuclear cells from the CNS were isolated as described in the “Methods” section from PFF α-syn or monomer α-syn mice at 5 months p.i. (*n* = 6 per group). **a** Gating strategy for flow cytometry data analysis. **b** Plots show relative percentages of CD19+ B cells, T cells (CD4+ and CD8+ T cells), CD11b+CD45^low^ (microglia), CD11b+CD45^high^ (macrophage or activated microglia), Ly6+ neutrophils, and NK1.1+ NK cells (blue: monomer α-syn; red: PFF α-syn). Data represent mean ± SEM. *p < 0.05, **p < 0.01, ***p < 0.001, n.s.; not significant, Student’s *t* test

To investigate the changes in immune cell composition and peripheral immune cell infiltration within the CNS parenchyma while LB-like inclusions were present in various brain regions, we euthanized mice at 5 months p.i. and perfused with buffered saline to remove immune cells in the blood vessels and isolated mononuclear immune cells in the CNS as we previously described [31]. We then determined the relative percentage of immune cell subsets by flow cytometry analysis. Based on our gating strategy shown in Fig. 4a, we observed significantly increased relative percentages of B lymphocytes (CD19+), T-helper lymphocytes (CD4+), T-cytotoxic lymphocytes (CD8+), activated myeloid cells (CD11b+ CD45^high^), and natural killer (NK) cells (TCRβ− NK1.1+), while the relative percentage of resting myeloid cells (CD11b+ CD45^low^) were decreased in PFF-inoculated mice compared with monomer controls (Fig. 4b). Our data demonstrate substantial peripheral immune cell infiltration into the brain of PFF α-syn treated mice at 5 months p.i. The relative percentage of neutrophils was not altered in PFF α-syn mice compared with monomer controls (Fig. 4b). Our data implicate that LB-like pathology in the CNS is associated with augmented immune cell infiltration prior to alterations in DA neurodegeneration in the mouse brain.

### PFF α-syn–injected mice display increased total number of immune cells in the spleen 5 months p.i.

**Fig. 5:**
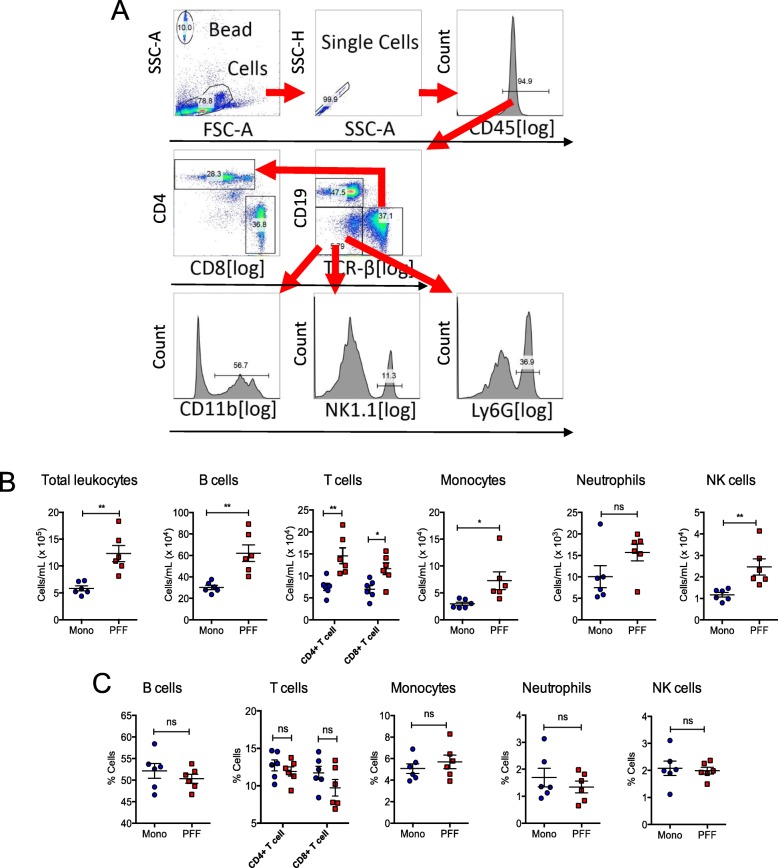
PFF α-syn–injected mice display increased total number of immune cells within the spleen. Mononuclear immune cells from the spleen were isolated from PFF α-syn or monomer α-syn mice at 5 months p.i. (*n* = 6 per group). **a** The gating strategy for lymph node, spleen, and blood flow cytometry data analyses. **b** Plots show the numbers of total CD45+ leukocytes, CD19+ B cells, CD4+ T cells, CD8+ T cells, CD11b+ macrophage, Ly6+ neutrophils, and NK1.1+ NK cells. **c** Plots show the percentages of CD19+ B cells, T cells (CD4+ and CD8+ T cells), CD11b+ macrophage, Ly6+ neutrophils, and NK1.1+ NK cells under the CD45+ gated leukocytes (blue: monomer α-syn; red: PFF α-syn). Data represent mean ± SEM. *p < 0.05 or **p < 0.01, n.s. not significant, Student’s *t* test

Based on our observations revealing exacerbated immune cell infiltration within the CNS in conjunction with p-α-syn positive inclusions within the ENS of PFF α-syn mice, we examined immune cell composition in the peripheral immune system. For that, we isolated total splenocytes and analyzed the immune cell profile by flow cytometry. Based on our gating strategy (Fig. 5a), we observed a statistically significant increase in total numbers of leukocytes within the spleens of PFF α-syn mice compared with monomer controls (Fig. 5b). Furthermore, we observed an increase in total number of B cells, CD4+ T cells, CD8+ T cells, monocytes, and NK cells in PFF α-syn mice compared with monomer controls at 5 months p.i. (Fig. 5b). However, no significant changes were observed in the relative percentage of immune cells in the spleen between monomer and PFF α-syn–injected animals (Fig. 5c). These results indicate that CNS-initiated synucleinopathies are associated with alterations in the total number of immune cells within secondary lymphoid organs, such as the spleen.

### Alterations in percentage and total number of immune cells in the inguinal lymph nodes of PFF α-syn mice

**Fig. 6:**
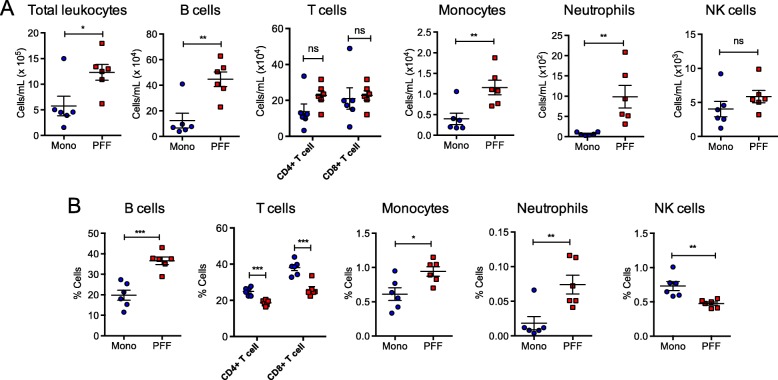
Alterations in percentage and total number of immune cells in the inguinal lymph node of PFF α-syn mice. Mononuclear immune cells from the inguinal lymph node were isolated from PFF α-syn or monomer α-syn mice at 5 months p.i. (*n* = 6 per group). **a** Plots show the numbers of total CD45+ leukocytes, CD19+ B cells, T cells (CD4+ and CD8+ T cells), CD11b+ macrophage, Ly6+ neutrophils, and NK1.1+ NK cells (blue: monomer α-syn; red: PFF α-syn). **b** Plots show the percentages of CD19+ B cells, CD4+ T cells, CD8+ T cells, CD11b+ macrophage, Ly6+ neutrophils, and NK1.1+ NK cells under the CD45+ gated leukocytes. Data represent mean ± SEM. Data represent mean ± SEM. *p < 0.05 or **p < 0.01, n.s.; not significant, Student’s *t* test

As the inguinal lymph nodes are another primary site of immune cell activation in the periphery [39], we isolated single cells from the inguinal lymph nodes and analyzed their immune cell contents through flow cytometry using the same gating strategy in Fig. 5a. We observed a statistically significant increase in the total number of leukocytes within the draining inguinal lymph nodes of PFF α-syn mice compared with monomer controls (Fig. 6a). Moreover, we observed an increase in total number of B cells, monocytes, and neutrophils in the PFF α-syn mice compared with monomer controls, but no changes in T cells or NK cells (Fig. 6a). We saw significant increases in the relative percentages of B cells, monocytes, and neutrophils but decreased percentages of T and NK cells in PFF α-syn mice compared with monomer controls (Fig. 6b). These results provide further evidence that intracerebral-initiated synucleinopathies promote changes in immune cell composition in areas of high immunoreactivity, such as the inguinal lymph nodes.

### PFF α-syn mice display no changes in percentage of immune cells in the blood but exhibit decreased total numbers of monocytes, neutrophils, and NK cells

**Fig. 7:**
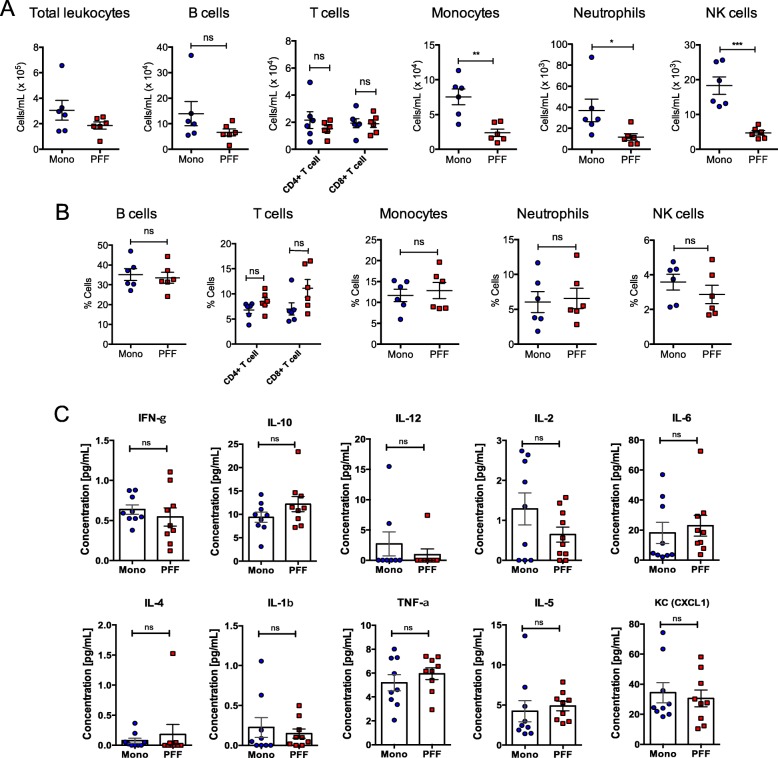
PFF α-syn mice display no changes in percentage of immune cells in the blood but exhibit decreased numbers of monocytes, neutrophils, and NK cells. Blood PBMCs were prepared from PFF α-syn or monomer α-syn mice at 5 months p.i. (*n* = 6 per group). **a** Plots show the numbers of total CD45+ leukocytes, CD19+ B cells, CD4+ T cells, CD8+ T cells, CD11b+ macrophage, Ly6+ neutrophils, and NK1.1+ NK cells. **b** Plots show the percentages of CD19+ B cells, T cells (CD4+ and CD8+ T cells), CD11b+ macrophage, Ly6+ neutrophils, and NK1.1+ NK cells under the CD45+-gated leukocytes (blue: monomer α-syn; red: PFF α-syn). **c** Plots show Multiplex proinflammatory cytokine analyses in serum. Data represent mean ± SEM. *p < 0.05, **p < 0.01, ***p < 0.001, n.s.; not significant, Student’s *t* test

To further characterize immune cell composition within the periphery, we isolated total leukocytes from the blood of mice 5 months after PFF or monomer α-syn intrastriatal injection and quantified the total number and relative percentage of immune cell subsets. We observed no significant changes in the total number of leukocytes including B cells and T cells, but saw decreased numbers of monocytes, neutrophils, and NK cells in the blood of PFF α-syn mice compared with monomer controls (Fig. 7a). Furthermore, no significant changes in the percentages of immune cell populations were observed in the blood of PFF α-syn mice compared with monomer controls (Fig. 7b). Next, we assessed changes in cytokine and chemokine content in serum, which act as soluble mediators of innate and adaptive immunity and are often involved in inflammation through their effects on antigen presentation, cell recruitment, cell activation, and adhesion molecule expression [40]. We performed a multiplex immune assay and demonstrated that there are no major changes in cytokines or chemokines in the serum of PFF α-syn mice compared with the monomer controls (Fig. 7c).

## Discussion

In the brain, microglia are able to uptake and degrade extracellular α-syn [41, 42], and present α-syn peptides to T cells to mount an immune response [36]. This may exacerbate the progression of neurodegeneration by enhancing the release of proinflammatory cytokines and increasing oxidative stress [43]. Mice that overexpress wild-type human α-syn display early microglia activation and high levels of proinflammatory cytokine production [44]. Furthermore, peripheral macrophages are recruited into α-syn fibril–injected rat brains prior to neurodegeneration [12]. In non-Tg mice inoculated with PFF α-syn, we showed for the first time that LB-like pathology led to robust microglial activation, astrogliosis, and increased infiltration of peripheral immune cells including B, CD4+T, CD8+ T, activated myeloid cells, and NK cells within the CNS prior to DA neurodegeneration.

Peripherally, α-syn aggregates were found within the myenteric plexus of the gut [45]. Furthermore, substantial increases of inflammatory cytokines (IL-1β, TNF-α, IL-6) were displayed in the gut of PD patients [46]. Macrophages within the gut have reduced phagocytic activity against α-syn aggregates, and intestinal permeability was correlated with the presence of α-syn aggregates in aged rat, [14] implicating immune responses specific for α-syn species in the periphery. Here, we found that CNS-initiated synucleinopathy in non-Tg mice was transmitted to the gut. We also demonstrated that PFF α-syn inoculated non-Tg mice display altered lymphocyte profiles in the periphery. Potential mechanisms by which CNS-initiated synucleinopathy altered the peripheral immune profiles are as follows: (1) In the CNS parenchyma, the drainage of cellular debris and waste products from cell metabolism via a paravascular route (the glymphatic route) of the meningeal lymphatic system through which interchange between the CSF and interstitial fluid (ISF) takes place [47–50]. The meningeal lymphatic system drains directly into the deep cervical lymph nodes [51] and acts as a route for drainage of macromolecules, such as the amyloid-beta (Aβ) protein seen in Alzheimer’s disease (AD) [52], which may attribute to the conventional lymphatic vasculature in the periphery as we observed the changes in peripheral immune phenotypes in our mouse model; (2) Another possible mechanism is the intimate connection of the gut-brain axis. The gut-brain axis allows correspondence between the CNS and periphery through the most direct path via the vagus nerve in the gut, which originates in the dorsal motor nucleus in the medulla and extends through the abdomen to the viscera [53]. It has been suggested that α-syn pathology may begin within the ENS and propagate up to midbrain regions through the vagal nerve afferents [35, 54, 55]. Recent study demonstrated that synucleinopathies initiated in the CNS propagate into the ENS in rat models of PD at 6 months p.i., supporting the propagation of synuclein between the brain and the periphery [34]. Immune cells are capable of engaging in direct communication with neurons through the vagal nerve [53, 56]. Therefore, the changes in peripheral immune phenotypes in our mouse model could be related to synuclein pathology propagating into the periphery as we saw within the ENS.

We note that this study is rather preliminary in scope and the mechanism by which the immune cell changes that occur in this model were not clearly elucidated. However, we provided primary information that extensive immune responses in the CNS and the periphery occur in PFF α-syn–injected non-Tg mice and these responses occur prior to neurodegeneration. A recent studies suggest cell type-specific immune responses may be associated with different forms of α-syn and with other pathological phenotypes in PD. Nissen et al. showed that monocytes from PD patients are highly proliferative when compared with those from healthy controls [57]. CD4+ T cells from PD patients exhibit immune responses to α-syn epitopes presented by MHC-II [58]. Furthermore, baseline levels and the inducibility of MHC-II in antigen presenting cells were heightened in PD patients [59]. Therefore, future studies aimed at interrogating alterations in immune cell specificity and functionality in PD could divulge the mechanism of altered immune cell profiles and the association of early alterations in immune responses and disease phenotypes. This study will allow others and us to utilize the PFF α-syn model in mice with genetic modifications to dissect the mechanisms of neuroinflammation and their effect on protein aggregation and PD pathologies.

## Conclusions

Here, we provide a primary characterization of immune phenotypes in PFF α-syn–injected non-Tg mice. PFF α-syn inoculation in non-Tg mice induced neuroinflammation, increased infiltration of peripheral immune cells in the CNS, and the propagation α-syn inclusions in the gut prior to DA neurodegeneration at 5 months p.i. Furthermore, we observed substantial alterations in immune cell profiles in peripheral lymphoid organs. Our study allows us to utilize this mouse model to assess the mechanistic link between immune responses and protein aggregation in PD or other neurological pathologies.

## Data Availability

All data generated or analyzed during this study are included in this published article

## Abbreviations

AA: Amino acid
AD: Alzheimer’s disease
BBB: Blood-brain barrier
CNS: Central nervous system
CSF: Cerebrospinal fluid
CXCL10: C-X-C chemokine motif 10
DA: Dopaminergic
DAMP: Damage-associated molecular pattern
ENS: Enteric nervous system
GFAP: Glial fibrillary acidic protein
HLA-DR: Human leukocyte antigen D related
Iba-1: Ionized calcium-binding adaptor molecule 1
IFN-γ: Interferon-gamma
IL-1β: Interleukin one-beta
IL-6: Interleukin-six
iNOS: Inducible nitric oxide synthase
ISF: Interstitial fluid
LB: Lewy Body
MHC-II: Major-histocompatibility complex-class two
NK cell: Natural killer cell
NO: Nitric oxide
NSAIDS: Non-steroidal anti-inflammatory drugs
PBMC: Peripheral blood mononuclear cell
PD: Parkinson’s disease
PFA: Paraformaldehyde
PFF: Preformed fibril
p-α-syn: PhosphoSerine129 alpha-synuclein
ROS: Reactive oxygen species
RT: Room temperature
SNpc: Substantia Nigra pars compacta
TH: Tyrosine hydroxylase
TLR-2: Toll-like receptor 2
TLR-4: Toll-like receptor 4
TNF-α: Tumor necrosis factor-alpha
α-syn: Alpha-synuclein
